# *Hirsutella sinensis* Attenuates Aristolochic Acid-Induced Renal Tubular Epithelial-Mesenchymal Transition by Inhibiting TGF-β1 and Snail Expression

**DOI:** 10.1371/journal.pone.0149242

**Published:** 2016-02-18

**Authors:** Xiao-yi Xu, Jing-jing Chai, Yi-pu Chen, Hong-liang Rui, Yan-yan Wang, Hong-rui Dong, Yu-lin Man, Hong Cheng

**Affiliations:** 1 Division of Nephrology, Beijing Anzhen Hospital, Capital Medical University, Beijing 100029, China; 2 Emergency Department, Peking Union Medical College Hospital, Beijing 100730, China; Institut National de la Santé et de la Recherche Médicale, FRANCE

## Abstract

**Objective:**

To investigate the inhibitory effect of *Hirsutella sinensis* (HS) on epithelial-mesenchymal transition (EMT) of renal tubular epithelial cells induced by aristolochic acid (AA) and its possible mechanism.

**Methods:**

18 male Sprague-Dawley rats were randomly and equally divided into the following 3 groups: AA group, AA+HS group and control group. Urinary protein excretion and creatinine clearance (CCr) were measured. All rats were sacrificed at the end of 12th week. The pathological examination of renal tissue was performed and the mRNA and protein expression of transforming growth factor-β1 (TGF-β1), α-smooth muscle actin (α-SMA), cytokeratin-18 and Snail in renal cortex were determined by real time quantitative PCR and immunohistochemical staining respectively. In addition, human renal proximal tubule epithelial cells line (HKC) was divided into the following 4 groups: AA group, AA+HS group, HS control group and control group. The above mRNA and protein expression in HKC was determined by real time quantitative PCR and Western blot respectively.

**Results:**

(1) CCr was significantly decreased, and the urinary protein excretion and relative area of renal interstitial fibrosis were significantly increased in the rats of AA and AA+HS group compared to those in control group (*P*<0.05 or *P*<0.01); all the above abnormalities significantly lightened in the rats of AA+HS group compared to those in AA group (*P*<0.05). (2) The mRNA and protein expression of TGF-β1, α-SMA and Snail was significantly up-regulated and the expression of cytokeratin-18 was significantly down-regulated in the rat renal cortex as well as in the cultured HKC cells in AA and AA+HS groups compared to those in control group (*P*<0.05 or *P*<0.01); all the above abnormalities significantly alleviated in AA+HS group compared to those in AA group (*P*<0.05 or *P*<0.01). (3) Knockdown endogenous Snail expression by siRNA could ameliorate AA-induced EMT of HKC cells, while overexpression of Snail by plasmid transfection diminished the antagonistic effect of HS on AA-induced EMT. These results suggest Snail might be a potential target of HS effect.

**Conclusion:**

HS is able to antagonize, to some extent, tubular EMT and renal interstitial fibrosis caused by AA, which might be related to its inhibitory effects on the TGF-β1 and Snail expression.

## Introduction

Renal interstitial fibrosis is the common pathway in progressive kidney disease, which leads to deterioration and eventual failure of renal function, irrespective of the diverse causes of disease [1,2]. The interstitial fibrosis process is often accompanied by epithelial-mesenchymal transition (EMT) of tubular epithelial cells, by which tubular epithelial cells are converted into the phenotype of myofibroblasts and produce interstitial matrix components [1–3]. Many studies have shown that transforming growth factor-β1 (TGF-β1) and its downstream transcription factor Snail are the key molecules that trigger the process of tubular EMT [1–5].

Our previous *in vitro* studies using cell culture techniques, including cell co-culture, have revealed that tubular epithelial cells can be activated by aristolochic acid (AA) and then secrete fibrogenic factors including TGF-β1, which can in turn act on renal interstitial fibroblasts through cell-cell cross talking to enhance collagen type I synthesis [6,7]. In addition, we have successfully created a rat model of chronic aristolochic acid nephropathy (CAAN) with typical interstitial fibrosis [8, 9] and demonstrated that EMT of tubular epithelial cells is involved in the development of interstitial fibrosis in the CAAN rat model *in vivo* [10]. Therefore, in the present study, we choose the cultured tubular epithelial cells stimulated by AA and the rat model of CAAN to investigate the antagonistic effects of *Hirsutella sinensis* (HS) on tubular EMT.

HS is the anamorph of *Cordyceps sinensis* [11]. According to the record in the “Pharmacopoeia of People’s Republic of China”, *Cordyceps sinensis* is a kind of fungus that belongs to Clavicipitaceae [11]. *Cordyceps sinesis*, as a traditional Chinese medicine herb, and its anamorph HS are widely used for kidney disease treatment in China and have been identified to have therapeutic effects on delaying the progression of renal function damage in patients with chronic kidney diseases [12,13]. Our previous *in vitro* and *in vivo* studies also have shown that HS is able to antagonize the fibrogenic actions of AA [14, 15]. However, so far, the underlying mechanism of the protective effects of HS has not been fully elucidated. Does HS have an inhibitory effect on EMT in its anti-fibrotic mechanism? If so, is there a change of Snail expression involved in this process? Therefore, the aim of this research project is to answer the above questions in order to improve the understanding of the HS anti-fibrotic mechanism.

## Materials and Methods

### Animals and Ethics Statement

Sprague-Dawley rats were purchased from Vital River Laboratory Animal Technology Co. (Beijing, China). Rats were maintained under specific-pathogen-free conditions in the animal facility at the Beijing Heart Lung and Blood Vessel Diseases Institute. The rats were given a standard rodent chow and water ad libitum. All animal care and experimental protocols complied with the US National Institutes of Health Guide for the Care and Use of Laboratory Animals (publication no. 85–23, 1996) and were approved by the Institutional Animal Care and Use Committee of Capital Medical University.

### Animal experiment

Eighteen male Sprague-Dawley rats with weight of 200±10g at the age of 8 weeks were randomly and equally divided into the following 3 groups: (1) CAAN model group: the rats intermittently received extract of *Aristolochia manshuriensis Kom* (AmK) by gavages in the morning. In the beginning 5 days, the gavage dosage of AmK equaled to AA 20 mg/kg•d, and then gavage was stopped for 9 days; afterward, AmK dosage was reduced to equivalent dose of AA 15 mg/kg•d and daily gavage of every other week was performed until the end of 12th week. The preparation of AmK extract was carried out according to our previously described method [8]. (2) Intervention group: besides receiving AmK as mentioned above, the rats were also administered HS (Zhongmei Huadong Pharmaceutical Co.) in a dose of 1.5 g/kg•d by gavage every afternoon. (3) Control group: the rats were given tap water by gavage every morning.

24h urine samples were collected for the measurement of urinary protein excretion every 4 weeks. The urinary protein was quantified using Bradford Protein Assay Kit (Beyotime Biotechnology). Serum creatinine (SCr) and urinary creatinine (UCr) were detected by the picric acid method with automatic biochemistry analyzer (Hitachi 7170) at 0 and 12th week, and creatinine clearance (CCr) was calculated according to the following formula: CCr (ml/min) = UCr (μmol/L) × urine volume (ml/min) / SCr (μmol/L). All the rats were sacrificed after anesthetized with pentobarbital at the end of 12th week. A part of kidney tissue was fixed in 4% neutral formaldehyde solution for pathological and immunohistochemical examinations, and another part of kidney tissue was rapidly reserved in liquid nitrogen for real time quantitative polymerase chain reaction (PCR) analysis.

### Experiment of HKC cells

Human kidney proximal tubular epithelial cell line, HKC, was purchased from the American Type Culture Collection (ATCC). The cells were cultured in DMEM/F12 media (Life technologies, USA) supplemented with 10% inactivated fetal bovine serum (Life technologies, USA) at 37°C in humidified air with 5% CO2. Cultured HKC cells were divided into the following 4 groups: (1) AA stimulation group: the cells were incubated with 10μmol/L AA (Sigma); (2) HS intervention group: incubated with 10 μmol/L AA and 10 mg/L HS (Zhongmei Huadong Pharmaceutical Co.); (3) HS control group: incubated with 10mg/L HS; (4) Control group: only incubated with media. After 12 h and 36 h of incubation, the cells were harvested for real time quantitative PCR analysis and Western blot assay, respectively.

### Experiment of siRNA interference of HKC cells

The HKC cells were transiently transfected with 4 μl human Snail siRNA (Santa Cruz, sc-38398) or 4 μl control siRNA-A (Santa Cruz, sc-37007) using Lipofectamine 2000 (Life technologies, USA) according to the manufacturer’s instruction. After that, the transfected cells were cultured in DMEM/F12 media (Life technologies, USA) with 10% inactivated fetal bovine serum (Life technologies, USA) for 24 h, and then incubated with or without AA (10 μmol/L, Sigma). After 12 h and 36 h of incubation, the transfected cells were harvested for real time quantitative PCR analysis and Western blot assay, respectively.

The experiment was grouped as follows: (1) Control group: non-transfected HKC cells were incubated with media; (2) AA stimulation group: non-transfected HKC cells were incubated with 10 μmol/L AA; (3) AA-stimulated Snail siRNA transfection group: HKC cells transfected with Snail siRNA were incubated with 10μmol/L AA; (4) AA-stimulated control siRNA-A transfection group: HKC cells transfected by control siRNA-A were incubated with 10μmol/L AA.

### Experiment of Snail overexpression of HKC cells

Cultured HKC cells were transiently transfected with 3μg pGV167-Snail or 3μg vector pGV167 (Shanghai Genechem Co.) using Lipofectamine 2000 (Life technologies, USA) according to the manufacturer’s instruction. After that, the transfected cells were cultured in DMEM/F12 media (Life technologies, USA) with 10% inactivated fetal bovine serum (Life technologies, USA) for 24 h, and then the cells were continuously incubated with media only, 10 μmol/L AA or 10 μmol/L AA and 10 mg/L HS, respectively. After 12 h or 36 h incubation the cells were harvested for real time quantitative PCR analysis and Western blot assay, respectively.

### Pathological examination

The kidney tissue was conventionally dehydrated, embedded, cut into 3 μm-thick sections and stained with Masson trichrome reagent for light microscopy. The tubulointerstitial images of renal cortex, which did not contain glomeruli and arterioles, in 15 random visual fields (×100 times) were separately taken, and then were analyzed by Motic Med 6.0 digital medical image analysis system. The relative renal interstitial fibrosis area (%) = renal interstitial green dye area/visual field area ×100%.

### Immunohistochemical staining

3 μm-thick tissue sections of renal cortex were microwave heating-induced epitope retrieval (95°C for 10 min). Rabbit anti-TGF-β1 polyclonal antibody (Santa Cruz, 1:150), rabbit anti-Snail polyclonal antibody (Abcam, 1:125), mouse anti-α-smooth muscle actin (α-SMA) monoclonal antibody (1:30) and mouse anti-cytokeratin-18 monoclonal antibody (Zhong Shan Golden Bridge Biotech, 1:50) were used as primary antibodies and incubated with renal tissue sections respectively at 4°C over night. Then, the immunostaining of TGF-β1, Snail and cytokeratin-18 was performed using the EnVision detection kit (Dako), and the immunostaining of α-SMA was carried out using labeled streptavidin- biotin (LSAB) method [10].

The immunohistochemical staining images of TGF-β1, α-SMA and cytokeratin-18 were analyzed using the method similar to the image analysis of aforementioned Masson staining. The relative tubulointerstitial positive area = tubulointerstitial brown dye area/visual field area × 100%. The images of Snail were analyzed by the following procedures: the tubulointerstitial images of renal cortex, which did not contain glomeruli and arterioles, in 20 random visual fields (×400 times) were separately taken under light microscopy, and then the number of tubular epithelial cells with Snail positive staining in each visual field was counted. The Snail positive cell number per square millimeter area (number/mm^2^) = positive cell number per visual field/area of per visual field.

### Reverse transcription and real time quantitative PCR

Total RNA was isolated from rat renal cortex tissue or HKC cells using Trizol reagent (BBI) following the manufacturer’s instructions. 2 μg total RNA from each sample was reverse-transcribed to cDNA with Moloney murine leukemia virus reverse transcriptase (Huamei Biotech). Gene-specific primers see S1 and S2 Tables, respectively. Real time quantitative PCR was performed using SYBR Green Realtime PCR Master Mix (TOYOBO) following the manufacturer’s instructions. The GAPDH and β-actin fragments were also amplified as the internal control genes for the animal and cellular experiments, respectively. The relative quantity of mRNA expression was calculated according to the formula 2^-(target gene Ct–control gene Ct)^×10^3^, in which Ct is threshold cycle number.

### Western blot assay

The HKC cells were lysed using RIPA buffer (ComWin Biotec Co) and then the cell lysate was boiled for 5 min. Equivalent amounts of boiled cell proteins were separated by electrophoresis on sodium dodecyl sulfate-polyacrylamide gel (SDS-PAGE) and transferred to nitrocellulose membranes (General Electric Co). After blocking with 5% skim milk in phosphate-buffered saline with 0.1% Tween 20 for 1 h, the membranes were incubated with primary antibody at 4°C over night, and then incubated with secondary antibody in room temperature for 1 h. Details regarding primary and secondary antibodies are listed in S3 Table. The blotted proteins were quantified using Odyssey Infrared Imaging System (LI-COR Biosciences). β-actin was used as an internal loading control and the relative expression level of each target protein was displayed as a ratio of target protein/β-actin protein. All the assays were performed at least in triplicate.

### Statistical Analysis

All the data of continuous variables were expressed as the mean ± SD and analyzed by using SPSS 15.0 statistical software. One-way ANOVA was used for the comparison of multiple continuous variables. Correlation between two variables was inspected by using the Pearson correlation analysis. P<0.05 was considered statistically significant.

## Results

### The effects of HS on rat kidney injury caused by AA

**Fig 1:**
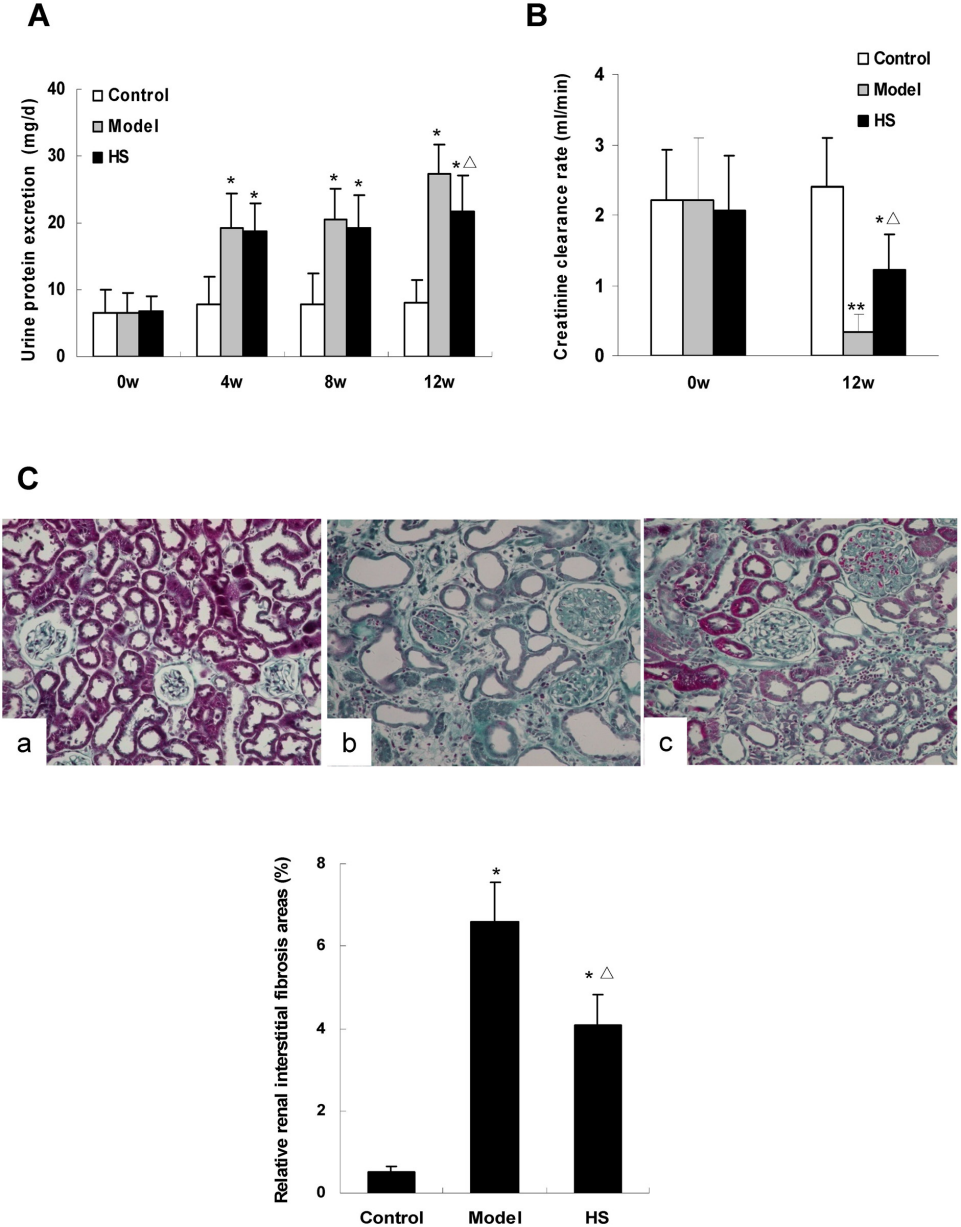
Effects of HS on urine protein excretion, creatinine clearance rate and renal interstitial fibrosis area of rat CAAN model. A: urine protein excretion of rats in control, CAAN model and HS intervention groups. B: creatinine clearance rate (CCr) of rats in control, CAAN model and HS intervention groups. C: histology of renal cortex tissue from rats of control (a), CAAN model (b) and HS intervention (c) groups (Masson staining ×200). The green parts indicate interstitial fibrosis areas. Values are represented as mean ± SD (n = 6). *P<0.05 vs. control group, **P<0.01 vs. control group, ^**Δ**^*P*<0.05 vs. model group.

Urinary protein excretion at baseline between the three animal groups had no statistical difference (*P*>0.05). Compared with control group, the urinary protein excretion in CAAN model group and intervention group was significantly increased at 4th, 8th and 12th week (*P*<0.01). Compared with CAAN model group, the urinary protein excretion in intervention group was decreased at 4th, 8th and 12th week, and the difference reached statistical significance at 12th week (*P*<0.05) (Fig 1A).

CCr levels at baseline among the three animal groups were not statistically different (*P*>0.05). At the 12th week, CCr levels in CAAN model and intervention groups were significantly lower than that in control group (*P*<0.05 or *P*<0.01), and in intervention group was significantly higher than that in CAAN model group (*P*<0.05) (Fig 1B).

Pathological examination of kidney tissue at the 12th week showed that the relative renal interstitial fibrosis areas in CAAN model and intervention groups were significantly larger than that in control group (*P*<0.05), and in intervention group was significantly smaller than that in CAAN model group (*P*<0.05) (Fig 1C).

The above results suggest that HS has antagonism effects on AA-induced kidney injury *in vivo*.

### Effects of HS on the expression of α-SMA and cytokeratin-18 in rat renal cortex tissues

**Fig 2:**
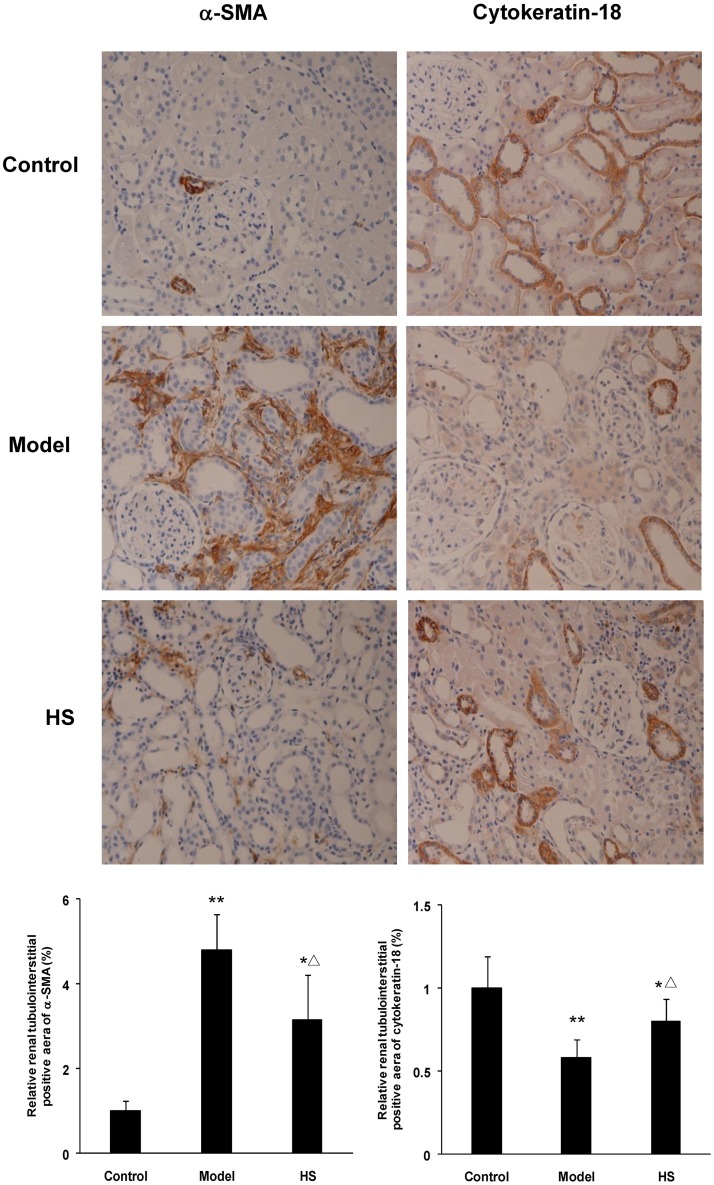
Effects of HS on protein expression of α-SMA and cytokeratin-18 in rat CAAN model. Immunohistochemistry for α-SMA and cytokeratin-18 in renal cortex tissues from control, CAAN model and HS groups. Magnification ×200. Protein expression of α-SMA and cytokeratin-18 was semi-quantitatively analyzed by image analysis system. Values are represented as mean ± SD (n = 6). *P<0.05 vs. control group, **P<0.01 vs. control group, ^**Δ**^*P*<0.05 vs. model group.

Immunohistochemical staining of rat renal cortex tissue displayed that α-SMA mainly expressed on arteriolar smooth muscle cells in control group, but also on tubular epithelial cells and in interstitium in CAAN model and intervention groups; cytokeratin-18 only expressed on tubular epithelial cells in the three groups (Fig 2).

**Fig 3:**
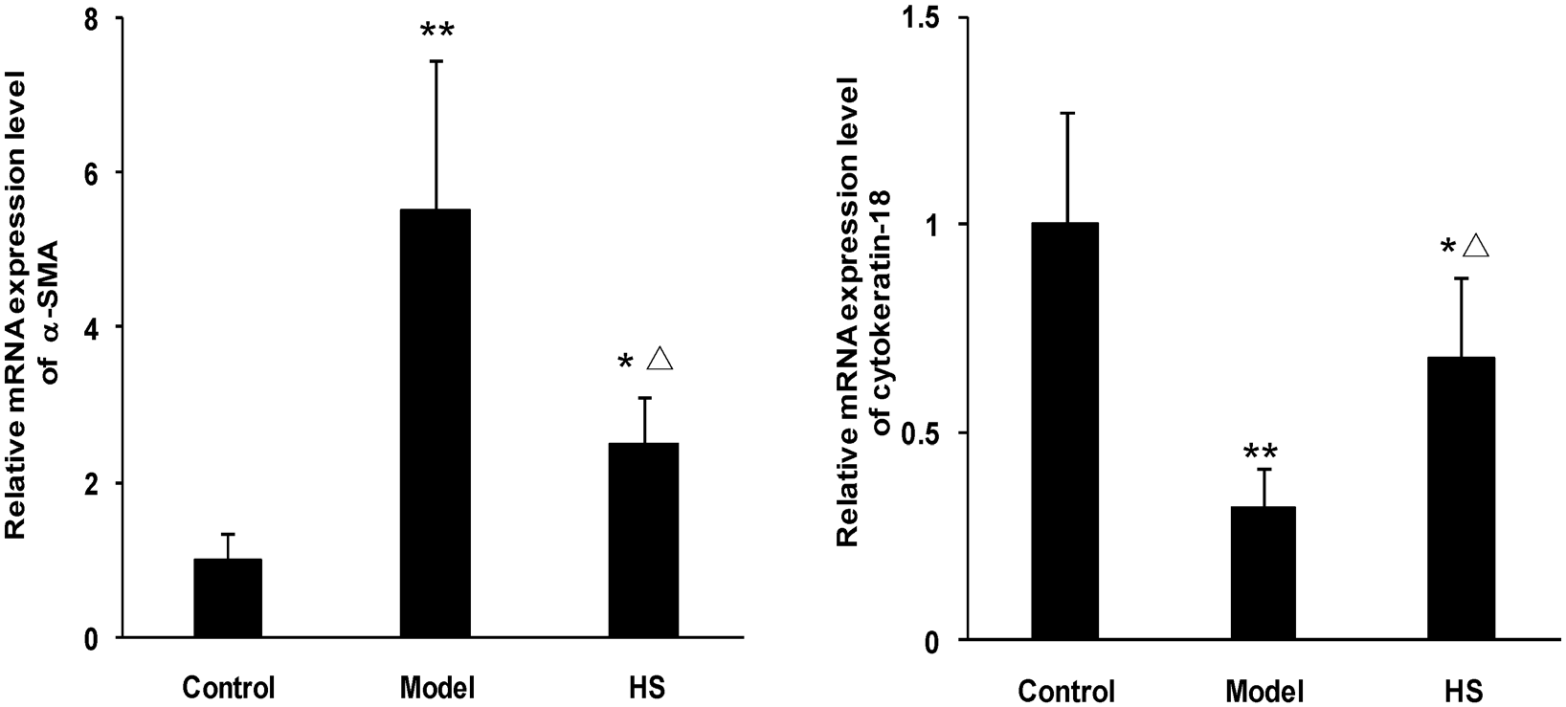
Effects of HS on α-SMA and cytokeratin-18 mRNA expression of rat CAAN model. Total RNA was extracted from renal cortex tissues and the relative mRNA expression levels of α-SMA and cytokeratin-18 were measured by real time quantitative PCR. Values are represented as mean ± SD (n = 6). *P<0.05 vs. control group, **P<0.01 vs. control group, ^**Δ**^*P*<0.05 vs. model group.

The animal experiment showed that α-SMA mRNA and protein expression in renal cortex tissue was significantly up-regulated in CAAN model and intervention groups compared with control group (*P*<0.05 or *P*<0.01), and was significantly down-regulated in intervention group compared with CAAN model group (*P*<0.05) (Figs 2 and 3).

In contrast, cytokeratin-18 mRNA and protein expression in renal cortex tissue was significantly down-regulated in CAAN model and intervention groups compared with control group (*P*<0.05 or *P*<0.01), and was significantly up-regulated in intervention group compared with CAAN model group (*P*<0.05) (Figs 2 and 3).

The above results suggest that the EMT of tubular epithelial cells occurs in the disease process of CAAN, and HS can antagonize the tubular EMT.

### Effects of HS on the expression of TGF-β1 and Snail in rat renal cortex tissues

**Fig 4:**
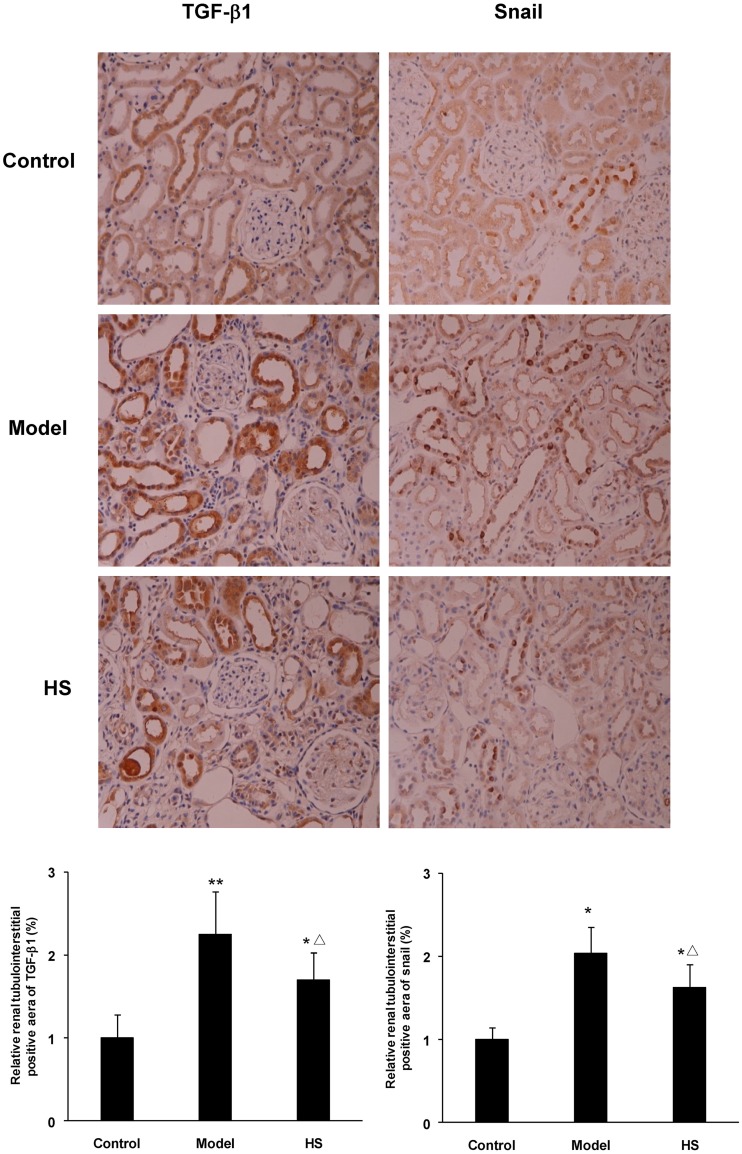
Effects of HS on protein expression of TGF-β1 and Snail in rat CAAN model. Immunohistochemistry for TGF-β1 and Snail in renal cortex tissues from control, CAAN model and HS groups. Magnification ×200. Protein expression of TGF-β1 and Snail was semi-quantitatively analyzed by image analysis system. Values are represented as mean ± SD (n = 6). *P<0.05 vs. control group, **P<0.01 vs. control group, ^Δ^*P*<0.05 vs. model group.

Immunohistochemical staining of rat renal cortex tissue in the three experimental groups displayed that TGF-β1 mainly expressed on tubular epithelial cells and weakly in glomeruli and arteriolar wall; Snail expressed in tubular epithelial cells (Fig 4).

**Fig 5:**
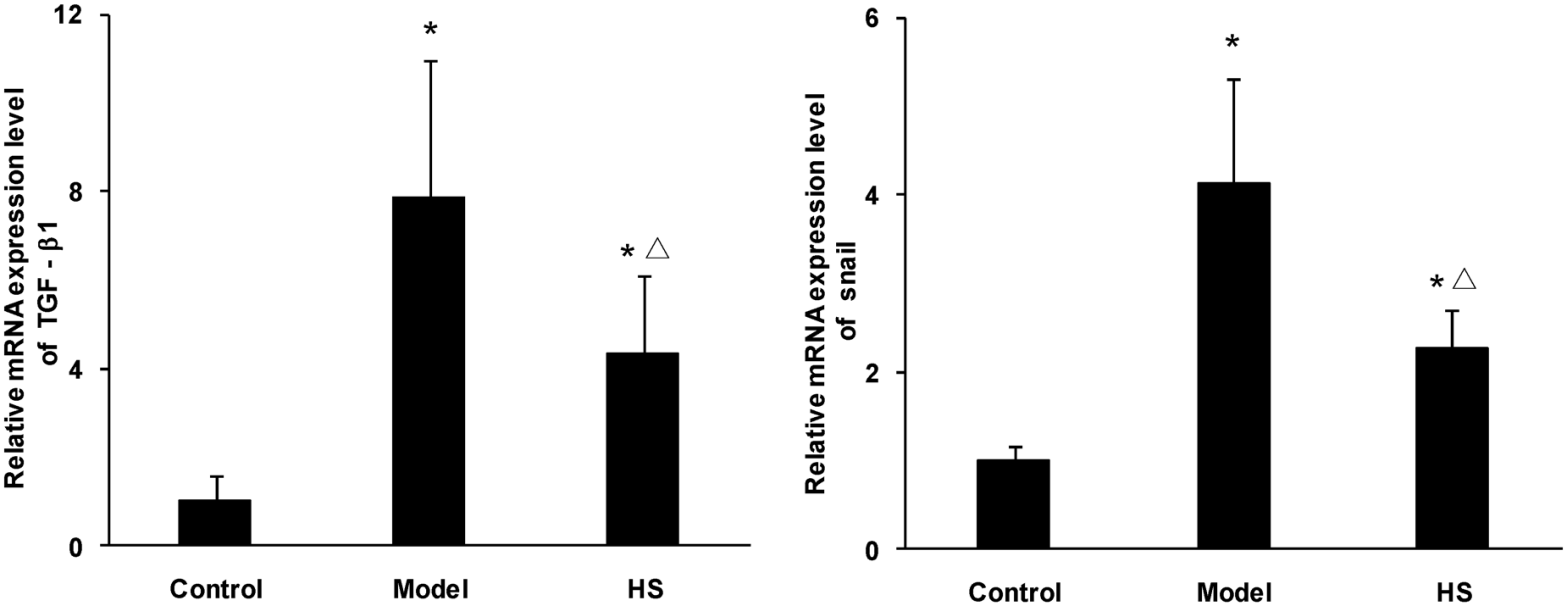
Effects of HS on TGF-β1 and Snail mRNA expression of rat CAAN model. Total RNA was extracted from renal cortex tissues and the relative mRNA expression levels of TGF-β1 and Snail were measured by real time quantitative PCR. Values are represented as mean ± SD (n = 6). *P<0.05 vs. control group, ^**Δ**^*P*<0.05 vs. model group.

The animal experiment showed that mRNA and protein expression of the TGF-β1 and Snail in renal cortex tissue was significantly up-regulated in CAAN model and intervention groups compared with control group (*P*<0.05 or *P*<0.01), and was significantly down-regulated in intervention group compared with CAAN model group (*P*<0.05) (Figs 4 and 5).

The above results suggest that there is the up-regulation of TGF-β1 and Snail expression in tubular epithelial cells in the CAAN rat model during the same time of tubular EMT, and HS can antagonize the effects.

### The correlation analyses between various parameters in CAAN rat model

The results of immunohistochemical staining on rat renal cortex tissues showed that the expression of TGF-β1 was positively correlated with expression of Snail and α-SMA, and negatively correlated with expression of cytokeratin-18 (correlation coefficients 0.715, 0.739 and -0.696, respectively. *P*<0.01); the expression of Snail was positively correlated with expression of α-SMA and negatively correlated with expression of cytokeratin-18 (correlation coefficients 0.843 and -0.740, respectively. *P*<0.01); the expression of α-SMA was negatively correlated with expression of cytokeratin-18 (correlation coefficient -0.735. *P*<0.01).

The results of Masson trichrome staining and immunohistochemical staining on rat renal cortex tissues showed that the relative renal interstitial fibrosis area was positively correlated with the expression of TGF-β1, Snail and α-SMA, and negatively correlated with the expression of cytokeratin-18 (correlation coefficients 0.787, 0.805, 0.915 and -0.814, respectively. *P*<0.01).

The above results suggest that the up-regulation of TGF-β1 and Snail expression in tubular epithelial cell is correlated with tubular EMT and renal interstitial fibrosis in the CAAN rat model.

### Effects of HS on the expression of α-SMA and cytokeratin-18 in cultured HKC

**Fig 6:**
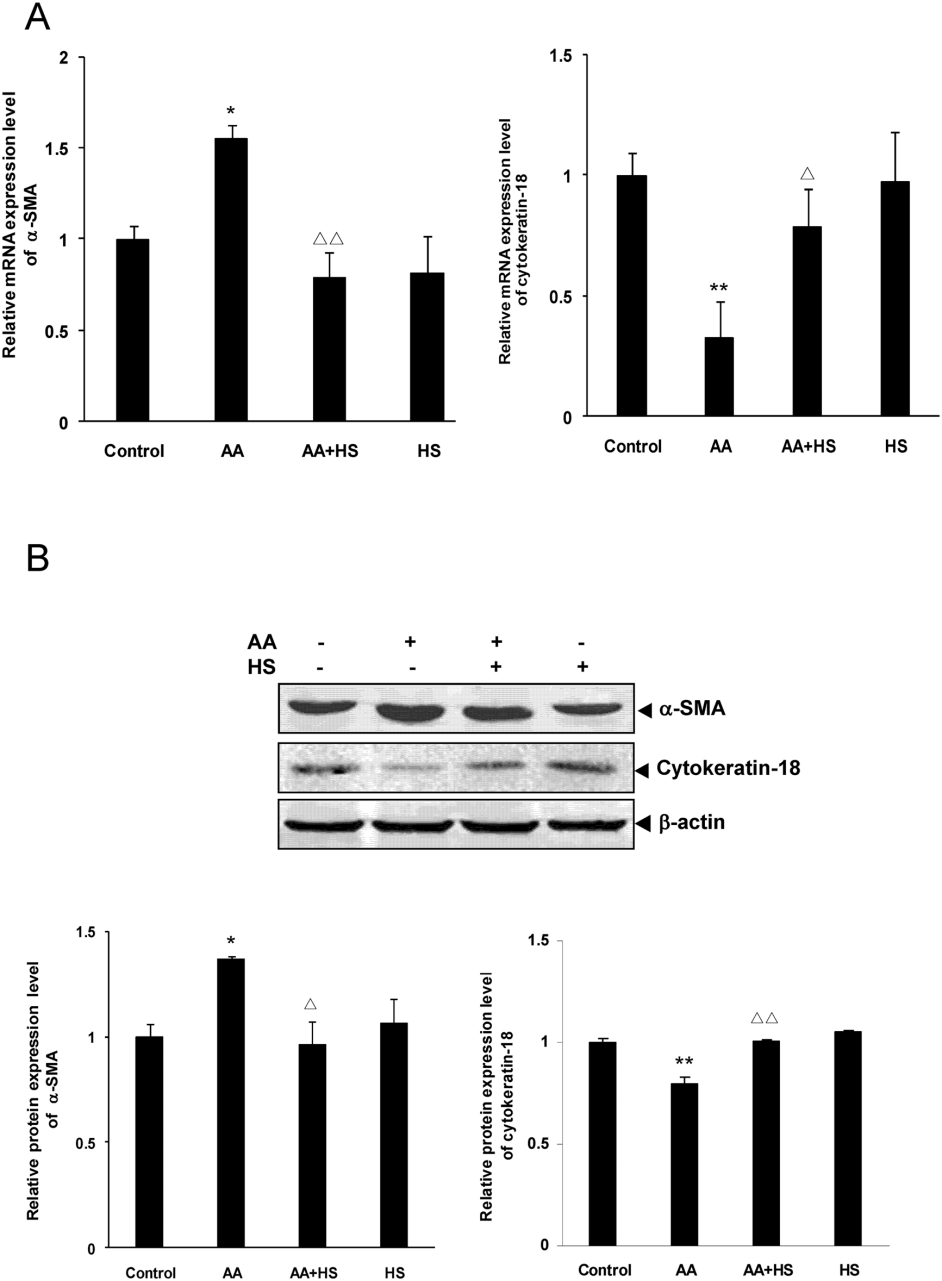
Effects of HS on AA-induced α-SMA and cytokeratin-18 expression in HKC cells. Cultured HKC cells were incubated in media, media containing 10 μmol/L AA and/or 10 mg/L HS, respectively. A: after 12 h of incubation, cells were collected and the mRNA expression levels of α-SMA and cytokeratin-18 were measured by real time quantitative PCR. B: after 36 h of incubation, cells were lysed and the total lysates were used to detect the protein expression levels of α-SMA and cytokeratin-18 by Western blot assay. The relative protein expression level was expressed as the target protein/β-actin protein ratio. Values are represented as mean ± SD (n = 3). *P<0.05 vs. control, **P<0.01 vs. control, ^Δ^*P*<0.05 vs. AA alone, ^ΔΔ^*P*<0.01 vs. AA alone.

The HKC cell experiment showed that α-SMA mRNA and protein expression was significantly up-regulated in AA stimulation group compared with control group (*P*<0.05), and was significantly down-regulated in HS intervention group compared with AA stimulation group (*P*<0.05 or *P*<0.01) (Fig 6A and 6B).

On the contrary, cytokeratin-18 mRNA and protein expression in HKC cells was significantly down-regulated in AA stimulation group compared with control group (P<0.01), and was significantly up-regulated in HS intervention group compared with AA stimulation group (P<0.05 or P<0.01) (Fig 6A and 6B).

The results of cell experiment are quite similar to those in animal experiment, and both suggest that HS can antagonize the tubular EMT caused by AA.

### Effects of HS on the expression of TGF-β1 and Snail in cultured HKC

**Fig 7:**
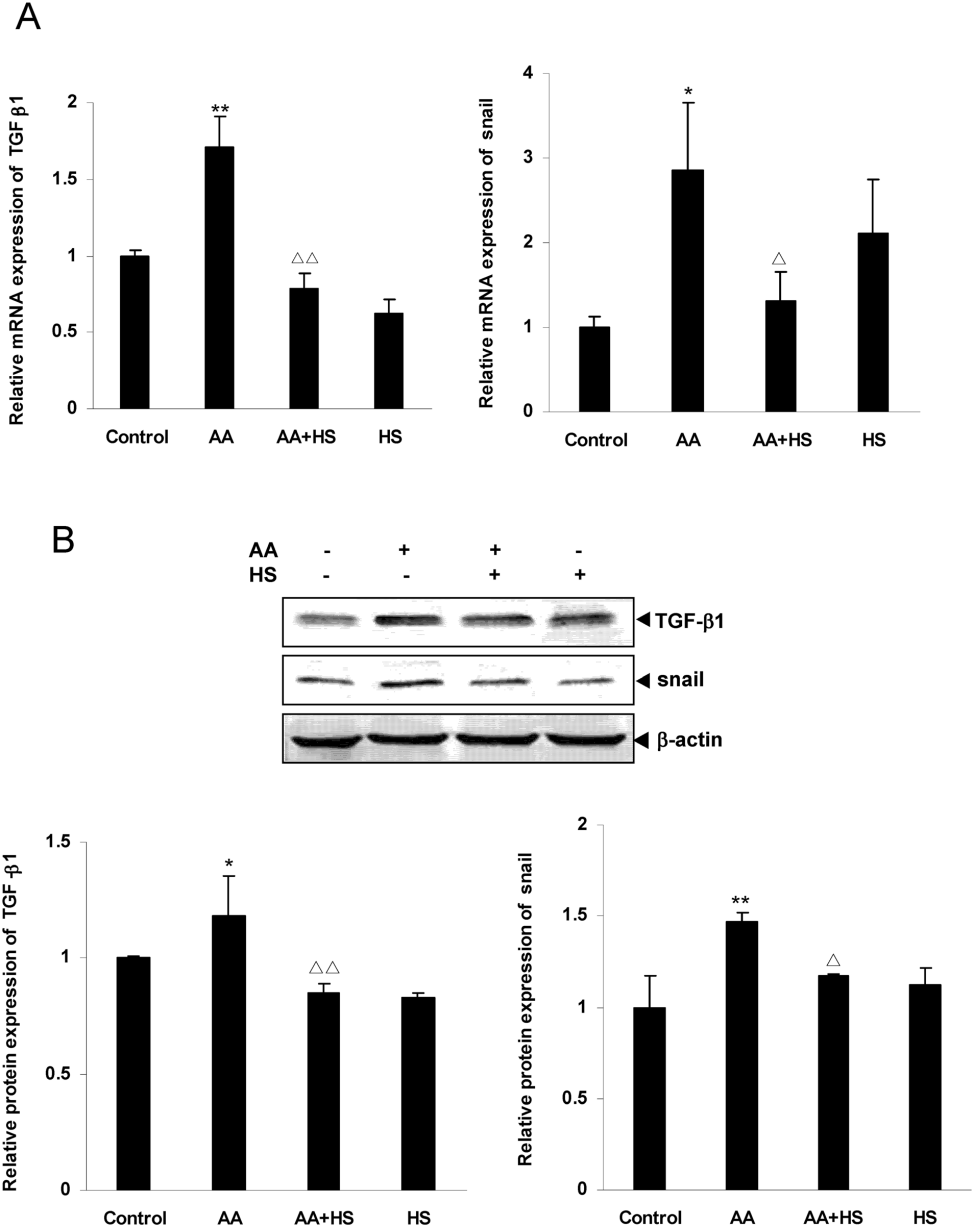
Effects of HS on AA-induced TGF-β1 and Snail expression in HKC cells. Cultured HKC cells were incubated in media, media containing 10 μmol/L AA and/or 10 mg/L HS, respectively. A: after 12 h of incubation, cells were harvested and the mRNA expression levels of TGF-β1 and Snail were measured by real time quantitative PCR. B: after 36 h of incubation, cells were lysed and the total lysates were used to determine the protein expression levels of TGF-β1 and Snail by Western blot assay. The relative protein expression level was expressed as the target protein/β-actin protein ratio. Values are represented as mean ± SD (n = 3). *P<0.05 vs. control, **P<0.01 vs. control, ^Δ^*P*<0.05 vs. AA alone, ^ΔΔ^*P*<0.01 vs. AA alone.

The HKC cell experiments showed that mRNA and protein expression of TGF-β1 and Snail was significantly up-regulated in AA stimulation group compared with control group (*P*<0.05 or *P*<0.01), and was significantly down-regulated in HS intervention group compared with AA stimulation group (*P*<0.05 or *P*<0.01) (Fig 7A and 7B).

The results of cell experiment, which are quite similar to the results of animal experiment, suggest that HS can antagonize the up-regulation of TGF-β1 and Snail expression of HKC cells caused by AA.

### The expression changes of Snail, α-SMA, cytokeratin-18 and fibronectin in HKC cells of Snail gene knockdown

**Fig 8:**
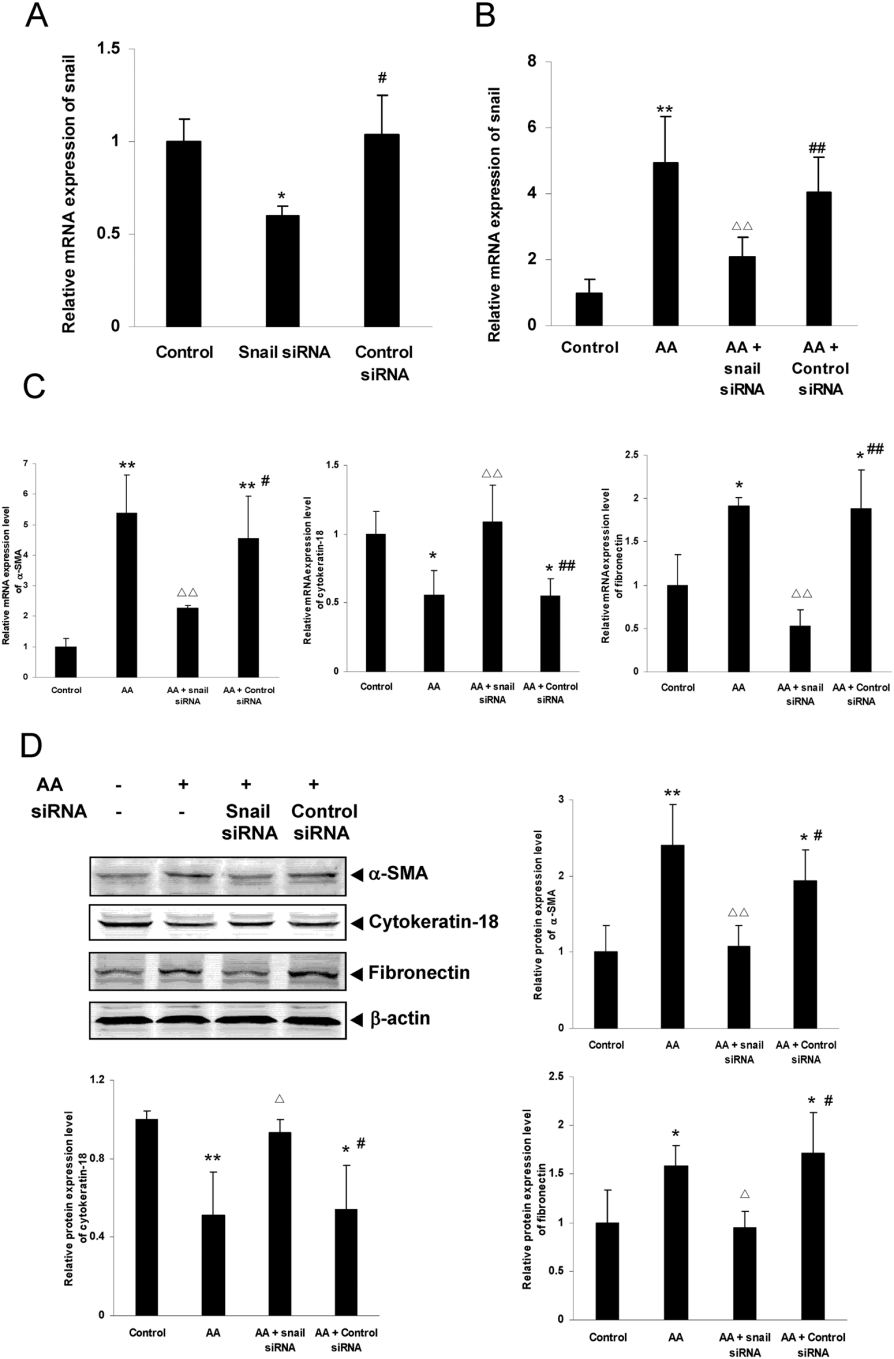
Effects of Snail gene knockdown on AA-induced Snail, α-SMA, cytokeratin-18 and fibronectin expression in HKC cells. HKC cells were transiently transfected with Snail siRNA or control siRNA. A: after transfection, mRNA expression of Snail was analyzed by real time quantitative PCR. B: after transfection, HKC cells were incubated with or without 10 μmol/L AA and then mRNA expression of Snail was analyzed by real time quantitative PCR. C and D: after transfection, HKC cells were incubated with or without 10 μmol/L AA. mRNA and protein expression of α-SMA, cytokeratin-18 and fibronectin were analyzed by real time quantitative PCR and Western blot assay respectively. Values are represented as mean ± SD (n = 3). *P<0.05 vs. control group, **P<0.01 vs. control group, ^Δ^*P*<0.05 vs. AA alone, ^ΔΔ^*P*<0.01 vs. AA alone, ^#^*P*<0.05 vs. Snail siRNA group, ^##^*P*<0.01 vs. Snail siRNA group.

Real time quantitative PCR analysis revealed that, compared with control group, the Snail mRNA expression was significantly down-regulated in Snail siRNA transfection group (*P*<0.05), but not changed in control siRNA-A transfection group (Fig 8A). The results suggest the siRNA transfection is successful.

The results of gene knockdown experiment showed that the mRNA and /or protein expression of Snail, α-SMA and fibronectin was significantly down-regulated (*P*<0.05 or *P*<0.01), and the mRNA and protein expression of cytokeratin-18 was significantly up-regulated (*P*<0.05 or *P*<0.01) in AA-stimulated Snail siRNA transfection group, compared with AA stimulation group. However, there were no similar changes of mRNA and protein expression in AA-stimulated control siRNA-A transfection group compared with AA stimulation group (Fig 8B, 8C and 8D).

These results, that down-regulation of Snail expression could significantly attenuate AA-induced EMT and matrix production of HKC cells, suggest that AA-induced fibrogenic actions might be mediated by transcription factor Snail.

### The expression changes of Snail, α-SMA, cytokeratin-18 and fibronectin in HKC cells of Snail overexpression

**Fig 9:**
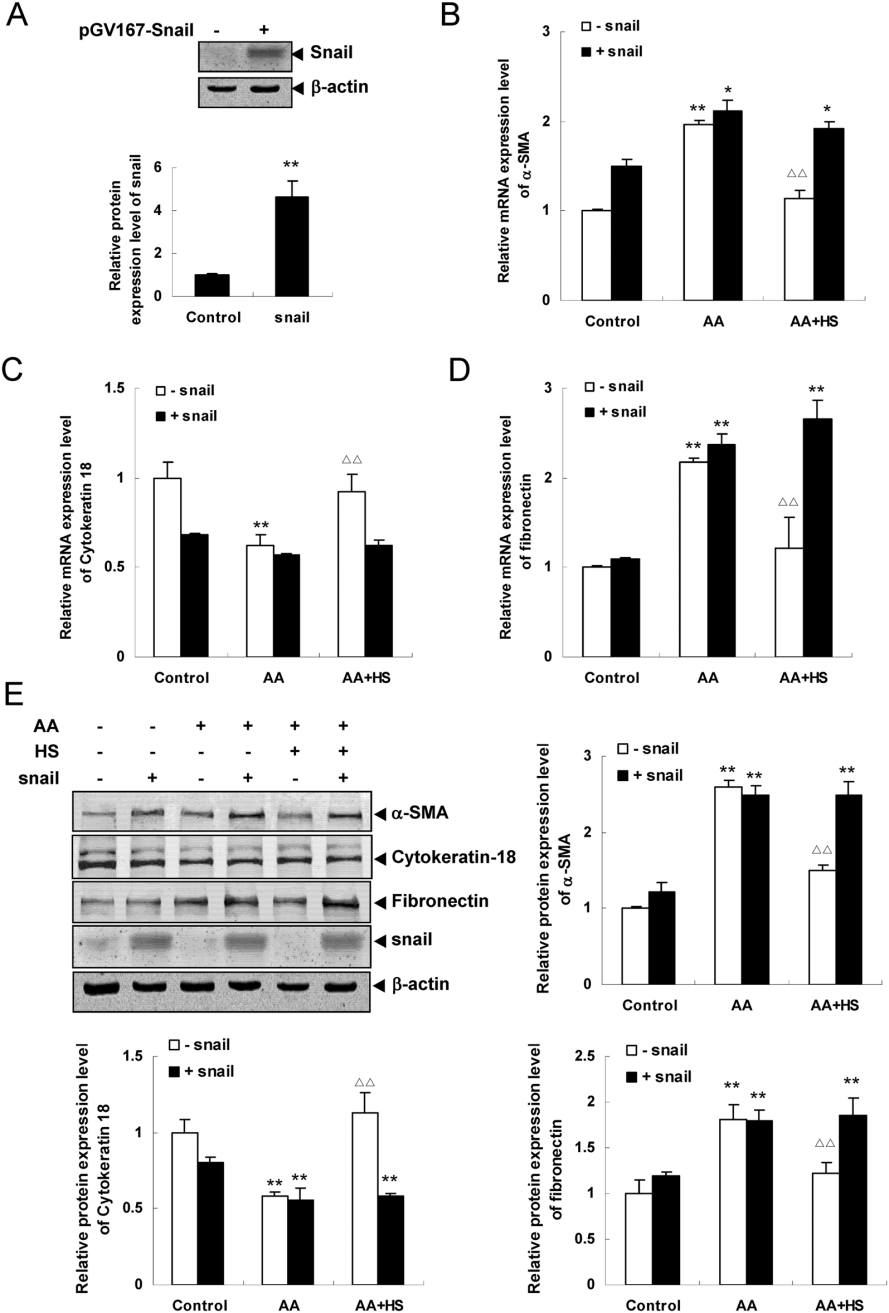
Effects of Snail overexpression on AA-induced Snail, α-SMA, cytokeratin-18 and fibronectin expression in HKC cells. HKC cells were transiently transfected with pGV167-Snail or pGV167 vector. A: after transfection, protein expression of snail was analyzed by Western blotting. B, C and D: after transfection, HKC cells were incubated with media alone, 10 μmol/L AA or 10 μmol/L AA and 10 mg/L HS, respectively. mRNA expression of α-SMA (B), cytokeratin-18 (C) and fibronectin (D) was analyzed by real time quantitative PCR. E: Protein expression of α-SMA, cytokeratin-18, fibronectin and Snail were analyzed by Western blot assay. The relative protein expression level was expressed as the target protein/β-actin protein ratio. Values are represented as mean ± SD (n = 3). *P<0.05 vs. control group, **P<0.01 vs. control group, ^ΔΔ^*P*<0.01 vs. AA alone.

Western blot assay revealed that, compared with control group, the Snail protein expression was significantly up-regulated in pGV167-Snail transfection group (*P*<0.01), but not changed in vector pGV167 transfection group (Fig 9A). The results suggest the pGV167-Snail transfection is successful.

The experimental results of Snail overexpression showed that the up-regulated mRNA and/or protein expression of Snail, α-SMA and fibronectin, and the down-regulated mRNA and protein expression of cytokeratin-18 were not able to inhabited by HS in AA-stimulated pGV167-Snail transfection group (*P*>0.05). However, the above changes of mRNA and/or protein expression were significantly reversed by HS in AA-stimulated vector pGV167 transfection group (*P*<0.05 or *P*<0.01) (Fig 9B to 9E).

These results, that HS could not weaken AA-induced EMT and matrix production in Snail overexpression HKC cells, suggest that the antagonistic effects of HS on AA-induced fibrogenic actions might be implemented by inhibiting Snail.

All original experimental data see S1 Data.

## Discussion

Myofibroblasts in renal interstitium, as predominant effector cells, play a vital role in the development of interstitial fibrosis by synthesizing and secreting extracellular matrix (ECM) [2]. During the past decades, the origins of renal interstitial myofibroblasts have been investigated extensively. Currently, they have been proposed to be derived from the following one or more sources: (1) activation of local resident fibroblasts; (2) differentiation of bone marrow-derived mesenchymal stem cells and fibrocytes; (3) transdifferentiation of pericytes; (4) tubular EMT; and (5) endothelial-mesenchymal transition [16–20]. Although the understanding has improved, the relative contribution of different cell sources to renal interstitial myofibroblasts remains controversial, especially for tubular EMT [19, 20]. Lebleu and colleagues reported that the cells of tubular epithelial origin undergoing EMT only contributed 5% to the renal interstitial myofibroblast pool [17,18], but this result could not be reproduced by subsequent studies [19], and the contribution of EMT even reached 36% in a previous report [21]. However, tubular EMT has been confirmed by many independent studies and its role in renal interstitial fibrosis is undisputed [19–21]. Therefore, we choose tubular EMT as the focus of this investigation to study the antagonistic effects of HS on renal interstitial fibrosis.

*Cordyceps sinensis*, also known by the Chinese name of *Dong Chong Xia Cao*, is a precious Chinese herbal medicine [12]. *Cordyceps sinensis* as a medicinal herb was first recorded in the Chinese classical medicine book “Ben Cao Bei Yao” (“Essentials of Materia Medica”) in 1694 [22], and its full scientific name is “*Cordyceps sinensis* (Berkeley) Saccardo,” named by Italian scholar Saccardo in 1878 [22]. HS is the anamorph of *Cordycep sinensis* [23, 24], which was verified by the studies of individual developmental biology [25] and molecular systems biology [26–28]. *Cordycep sinesis* and its anamorph HS are widely used to treat kidney diseases in China [12, 13]. Our previous *in vitro* and *in vivo* studies have shown that HS is able to antagonize the fibrogenic actions of AA by down-regulating the expression of TGF-β1, connective tissue growth factor (CTGF), plasminogen activator inhibitor-1 (PAI-1) and tissue inhibitor of metalloproteinases-1 (TIMP-1), which can promote ECM synthesis and/or inhibit ECM degradation [14,15]. However, the effect of HS on tubular EMT in renal interstitial fibrosis has not been investigated. In the process of EMT, the phenotypic conversion of tubular epithelial cells takes place. Cells lose epithelial cell markers such as E-cadherin and cytokeratin, acquire myofibroblastic markers such as α-SMA and vimentin, and produce interstitial matrix components including collagens type I and type III. In addition, the cell morphology and function are altered. The cell shape transforms from a cobblestone-like to a spindle-like appearance, and the cells lose their polarity and adhesion properties and increase their migration and invasion abilities [2, 3, 29–32]. In this study, we observed changes in the mRNA and protein expression levels of α-SMA and cytokeratin-18 both *in vitro* and *in vivo*. Our results showed that AA could give rise to the down-regulation of cytokeratin-18 expression and *de novo* α-SMA expression of tubular epithelial cells during the fibrogenic process, while HS significantly antagonized the effects of EMT induced by AA.

TGF-β1 and its downstream zinc finger transcription factor Snail are the key molecules that trigger the process of tubular EMT [1–5, 33–38]. TGF-β1 binds with its type I and type II transmembrane receptors, and then the cytoplasmic latent signal transduction proteins Smad 2/3 are phosphorylated by the type I receptor serine kinase. Phosphorylated Smad 2/3 partner with Smad 4 and subsequently translocate into the nuclei and up-regulate Snail expression [3, 33, 36, 39]. Snail binds to specific DNA sequences (CANNTG, where N is any nucleotide), called E-boxes in the promoter of the E-cadherin gene, and then represses E-cadherin transcription [4, 35, 38, 39]. E-cadherin, as a major constituent of adherens-type junctions, plays an essential role in the assembly of the junctional complex, maintaining the structural integrity and polarity of epithelial cells [3, 38]. Down-regulation or loss of E-cadherin expression would immediately induce *de novo* α-SMA expression and cause an early event of EMT [3, 34, 35, 39].

In this study, we observed changes in the mRNA and protein expression levels of TGF-β1 and Snail in tubular epithelial cells, and analyzed the relationship between their expression and tubular EMT as well as renal interstitial fibrosis in an animal experiment, and ECM production in a cell experiment. Both the in vitro and in vivo research results showed that AA significantly up-regulated the expression of TGF-β1 and Snail, while HS significantly antagonized the above AA-induced fibrogenic effects. Statistical correlation analysis in the animal experiment revealed that the expression of TGF-β1 was positively correlated with the expression of Snail, and that the expression of TGF-β1 and Snail was negatively correlated with the expression of cytokeratin-18 and positively correlated with the expression of α-SMA as well as the relative renal interstitial fibrosis area.

In order to confirm the effects of Snail on EMT and ECM production of tubular epithelial cells, a siRNA transfection experiment of HKC cells was performed. Real time quantitative PCR analysis and Western blot assay showed that Snail gene knockdown significantly up-regulated the AA-induced low expression of cytokeratin-18 and down-regulated the AA-induced high expression of Snail, α-SMA and fibronectin. In addition, a pGV167-Snail transfection experiment of HKC cells was also carried out. Real time quantitative PCR analysis and Western blot assay showed that AA-induced high expression of Snail, α-SMA and fibronectin, and low expression of cytokeratin-18 were not able to reverse by HS in the Snail overexpression HKC cell. These experimental results suggest that transcription factor Snail might play an important role in AA-induced fibronenic actions and the antagonistic effects of HS might be realized by suppressing Snail expression.

In conclusion, HS, the anamorph of *Cordyceps sinensis*, is able to antagonize effectively AA-induced tubular EMT and renal interstitial fibrosis. Transcription factor Snail might be one of potential targets of HS effect.

## Supporting Information

S1 Data Original experimental data. (DOC)

S1 Table Primer sequences for real time quantitative RT-PCR analysis in animal experiment. (DOC)

S2 Table Primer sequences for real time quantitative RT-PCR analysis in cell experiment. (DOC)

S3 Table Primary and secondary antibodies for Western blot assay. (DOC)
