# Supplementary material for: Hirsutella sinensis Attenuates Aristolochic Acid-Induced Renal Tubular Epithelial-Mesenchymal Transition by Inhibiting TGF-β1 and Snail Expression
Source: PLoS One. 2016 Feb 18;11(2):e0149242. doi: 10.1371/journal.pone.0149242 (PMC4759455; doi:10.1371/journal.pone.0149242)
Supplement: S1 Data — (DOC) [file pone.0149242.s001.doc]

***Original Experimental Data of manuscript PONE-D-15-31728R1***

**Fig 1. Effects of HS on urine protein excretion, creatinine clearance rate and renal interstitial fibrosis area of rat CAAN model.**

A: urine protein excretion of rats in control, CAAN model and HS intervention groups. (mg/24h，x ± s)(n＝6)

|  | 0w | 4w | 8w | 12w |
| --- | --- | --- | --- | --- |
| Control group | 6.7 ±3.2 | 7.9±4.1 | 7.7 ±4.8 | 8.0±3.4 |
| Model group | 6.6±2.8 | 19.2 ±5.3* | 20.5± 4.7* | 27.3±4.4* |
| HS group | 6.8 ±2.2 | 18.9 ±4.1* | 19.2±5.0* | 21.8±5.3*^#^ |

**P*<0.05 vs. control group, **^#^***P*<0.05 vs. model group.

B: Creatinine clearance rate (CCr) of rats in control, CAAN model and HS intervention groups. (ml/min，x ± s)(n＝6)

|  | 0w | 12w |
| --- | --- | --- |
| Control group | 2.22±0.70 | 2.39±0.71 |
| Model group | 2.22±0.87 | 0.33±0.25** |
| HS group | 2.07±0.78 | 1.22±0.51*^#^ |

**P*<0.05 vs. control group, ***P*<0.01 vs. control group, **^#^***P*<0.05 vs. model group.

C: Histology of renal cortex tissue from rats of control, CAAN model and HS intervention groups (%，x ± s) (n＝6)

|  | Interstitial fibrosis areas |
| --- | --- |
| Control group | 0.52±0.13 |
| Model group | 6.59±0.97* |
| HS group | 4.07±0.75*^#^ |

**P*<0.05 vs. control group, **^#^***P*<0.05 vs. model group.

**Fig 2. Effects of HS on protein expression of α-SMA and cytokeratin-18 in rat CAAN model. Immunohistochemistry for α-SMA and cytokeratin-18 in renal cortex tissues from control, CAAN model and HS groups. Magnification ×200. Protein expression of α-SMA and cytokeratin-18 was semi-quantitatively analyzed by image analysis system. (%,x ± s, n=6)**

|  | α-SMA | CK-18 |
| --- | --- | --- |
| Control group | 1±0.24 | 1±0.19 |
| Model group | 4.82±0.82** | 0.56±0.11** |
| HS group | 3.15±1.06*^#^ | 0.80±0.14*^#^ |

**P*<0.05 vs. control group, ***P*<0.01 vs. control group, ^#^*P*<0.05 vs. model group.

**Fig 3. Effects of HS on** **α-SMA and cytokeratin-18 mRNA expression of rat CAAN model.** Total RNA was extracted from renal cortex tissues and the relative mRNA expression levels of α-SMA and cytokeratin-18 were measured by real time quantitative PCR. (x ± s, n=6)

|  | α-SMA | CK-18 |
| --- | --- | --- |
| Control group | 1±0.35 | 1 ±0.27 |
| Model group | 5.52±1.96** | 0.32±0.10** |
| HS group | 2.49±0.60*^#^ | 0.68±0.20*^#^ |

**P*<0.05 vs. control group, ***P*<0.01 vs. control group, ^#^*P*<0.05 vs. model group.

**Fig 4. Effects of HS on protein expression of TGF-β1 and Snail in rat CAAN model.** Immunohistochemistry for TGF-β1 and Snail in renal cortex tissues from control, CAAN model and HS groups. Magnification ×200. Protein expression of TGF-β1 and Snail was semi-quantitatively analyzed by image analysis system. (x ± s, n=6)

|  | TGF-β1 (%) | Snail (cells/mm^2^) |
| --- | --- | --- |
| Control group | 1±0.28 | 1±0.14 |
| Model group | 2.24±0.52** | 2.03±0.32* |
| HS group | 1.70±0.33*^#^ | 1.62±0.28*^#^ |

**P*<0.05 vs. control group, ***P*<0.01 vs. control group, ^#^*P*<0.05 vs. model group.

**Fig 5. Effects of HS on** **TGF-β1 and Snail mRNA expression of rat CAAN model.** Total RNA was extracted from renal cortex tissues and the relative mRNA expression levels of TGF-β1 and Snail were measured by real time quantitative PCR. (x ± s, n=6)

|  | TGF-β1 | Snail |
| --- | --- | --- |
| Control group | 1±0.59 | 1±0.15 |
| Model group | 7.86±3.11* | 4.13±1.18* |
| HS group | 4.34±1.76*^#^ | 2.27±0.42*^#^ |

**P*<0.05 vs. control group, ^#^*P*<0.05 vs. model group.

Fig 6. Effects of HS on AA-induced α-SMA and cytokeratin-18 expression in HKC cells. Cultured HKC cells were incubated in media, media containing 10 μmol/L AA and/or 10 mg/L HS, respectively.

A: After 12 h of incubation, cells were collected and the mRNA expression levels of α-SMA and cytokeratin-18 were measured by real time quantitative PCR. (x± s，n=3)

|  | α-SMA | CK-18 |
| --- | --- | --- |
| Control | 1±0.07 | 1±0.09 |
| AA | 1.55±0.07* | 0.32±0.15** |
| AA+HS | 0.79±0. 14^##^ | 0.78±0.16^#^ |
| HS | 0.81±0.20 | 0.97±0.21 |

**P*<0.05 vs. control, ***P*<0.01 vs. control, ^#^*P*<0.05 vs. AA alone,

^##^*P*<0.01 vs. AA alone.

B: After 36 h of incubation, cells were lysed and the total lysates were used to detect the protein expression levels of α-SMA and cytokeratin-18 by Western blot assay. The relative protein expression level was expressed as the target protein/β-actin protein ratio (x ± s，n=3).

|  | α-SMA | CK-18 |
| --- | --- | --- |
| Control | 1±0.06 | 1±0.02 |
| AA | 1.37±0.01* | 0.79±0.03** |
| AA+HS | 0.96±0.12^#^ | 1.01±0.01^##^ |
| HS | 1.05±0.01 | 1.06±0.01 |

**P*<0.05 vs. control, ***P*<0.01 vs. control, ^#^*P*<0.05 vs. AA alone,

^##^*P*<0.01 vs. AA alone.

Fig 7. Effects of HS on AA-induced TGF-β1 and Snail expression in HKC cells. Cultured HKC cells were incubated in media, media containing 10 μmol/L AA and/or 10 mg/L HS, respectively.

A: After 12 h of incubation, cells were harvested and the mRNA expression levels of TGF-β1 and Snail were measured by real time quantitative PCR. (x ± s, n＝3)

|  | TGF-β1 | Snail |
| --- | --- | --- |
| Control | 1±0.04 | 1±0.13 |
| AA | 1.71±0.20** | 2.86±0.80* |
| AA+HS | 0.79±0.10^##^ | 1.31±0.35^#^ |
| HS | 0.62±0.09 | 2.1±0.64 |

**P*<0.05 vs. control, ***P*<0.01 vs. control, ^#^*P*<0.05 vs. AA alone,

^##^*P*<0.01 vs. AA alone.

B: After 36 h of incubation, cells were lysed and the total lysates were used to determine the protein expression levels of TGF-β1 and Snail by Western blot assay. The relative protein expression level was expressed as the target protein/β-actin protein ratio. (x ± s, n＝3)

|  | TGF-β1 | Snail |
| --- | --- | --- |
| Control | 1±0.01 | 1±0.18 |
| AA | 1.18±0.17* | 1.47±0.05** |
| AA+HS | 0.85±0.03^##^ | 1.17±0.01^#^ |
| HS | 0.83±0.02 | 1.12±0.09 |

**P*<0.05 vs. control, ***P*<0.01 vs. control, ^#^*P*<0.05 vs. AA alone,

^##^*P*<0.01 vs. AA alone.

Fig 8. Effects of Snail gene knockdown on AA-induced Snail, α-SMA, cytokeratin-18 and fibronectin expression in HKC cells. HKC cells were transiently transfected with Snail siRNA or control siRNA.

A: After transfection, mRNA expression of Snail was analyzed by real time quantitative PCR.

|  | **Snail** |
| --- | --- |
| Control | 1±0.12 |
| Snail siRNA | 0.60±0.05* |
| control siRNA | 1.05±0.21^#^ |

**P*<0.05 vs. control group, ^#^*P*<0.05 vs. Snail siRNA group.

B: After transfection, HKC cells were incubated with or without 10 μmol/L AA and then mRNA expression of Snail was analyzed by real time quantitative PCR. (x ± s, n=3)

|  | **Snail** |
| --- | --- |
| Control | 1±0.38 |
| AA | 4.86±1.38** |
| AA＋Snail siRNA | 2.05±0.59^△△^ |
| AA+control siRNA | 4.03±1.05^##^ |

***P*<0.01 vs. control group, ^△△^*P*<0.01 vs. AA alone,

^##^*P*<0.01 vs. Snail siRNA group.

C and D: after transfection, HKC cells were incubated with or without 10 μmol/L AA. mRNA and protein expression of α-SMA, cytokeratin-18 and fibronectin were analyzed by real time quantitative PCR and Western blot assay respectively. (x ± s, n=3)

|  | α-SMA | CK-18 | fibronectin |
| --- | --- | --- | --- |
| Control | 1±0.29 | 1±0.16 | 1±0.35 |
| AA | 5.38±1.27** | 0.56±0.18* | 1.92±0.10* |
| AA＋Snail siRNA | 2.26±0.09^△△^ | 1.09±0.27^△△^ | 0.53±0.19^△△^ |
| AA+control siRNA | 4.56±1.38**^#^ | 0.55±0.13*^##^ | 1.89±0.44*^##^ |

**P*<0.05 vs. control group, ***P*<0.01 vs. control group,^△△^*P*<0.01 vs. AA alone, ^#^*P*<0.05 vs. Snail siRNA group, ^##^*P*<0.01 vs. Snail siRNA group.

|  | α-SMA | CK-18 | fibronectin |
| --- | --- | --- | --- |
| Control | 1±0.36 | 1±0.04 | 1±0.35 |
| AA | 2.41±0.55** | 0.51±0.22** | 1.58±0.21* |
| AA＋Snail siRNA | 1.08±0.31^△△^ | 0.93±0.07^△^ | 0.95±0.16^△^ |
| AA+ control siRNA | 1.94±0.41*^#^ | 0.54±0.22*^#^ | 1.72±0.42*^#^ |

**P*<0.05 vs. control group, ***P*<0.01 vs. control group, ^△^*P*<0.05 vs. AA alone,

^△△^*P*<0.01 vs. AA alone, ^#^*P*<0.05 vs. Snail siRNA group.

Fig 9. Effects of Snail overexpression on AA-induced Snail, α-SMA, cytokeratin-18 and fibronectin expression in HKC cells. HKC cells were transiently transfected with pGV167-Snail or pGV167 vector.

A: After transfection, protein expression of snail was analyzed by Western blotting. The relative protein expression level was expressed as the target protein/β-actin protein ratio. Values are represented as mean ± SD (n=3).

|  | **Snail** |
| --- | --- |
| Control | 1±0.02 |
| Snail transfection | 4.60±0.77** |

***P*<0.01 vs. control group

B, C and D: After transfection, HKC cells were incubated with media alone, 10 μmol/L AA or 10 μmol/L AA and 10 mg/L HS, respectively. mRNA expression of α-SMA (9B), cytokeratin-18 (9C) and fibronectin (9D) was analyzed by real time quantitative PCR. The relative protein expression level was expressed as the target protein/β-actin protein ratio. Values are represented as mean ± SD (n=3).

|  |  | α-SMA | CK-18 | Fibronectin |
| --- | --- | --- | --- | --- |
| - Sanil | Control | 1±0.02 | 1±0.09 | 1±0.00 |
|  | AA | 1.96±0.05** | 0.62±0.06** | 2.18±0.04** |
|  | AA+HS | 1.14±0.09^##^ | 0.92±0.10^##^ | 1.22±0.34^##^ |
| + Snail | Control | 1.50±0.08 | 0.68±0.01 | 1.09±0.02 |
|  | AA | 2.11±0.13* | 0.57±0.01 | 2.37±0.12** |
|  | AA+HS | 1.92±0.07* | 0.62±0.03 | 2.66±0.21** |

**P*<0.05 vs. control group, ***P*<0.01 vs. control group, ^##^*P*<0.01 vs. AA alone.

E: Protein expression of α-SMA, cytokeratin-18, fibronectin and Snail were analyzed by Western blot assay. The relative protein expression level was expressed as the target protein/β-actin protein ratio. Values are represented as mean ± SD (n=3)

|  |  | α-SMA | CK-18 | Fibronectin |
| --- | --- | --- | --- | --- |
| - Sanil | Control | 1±0.01 | 1±0.08 | 1±0.14 |
|  | AA | 2.59±0.09** | 0.58±0.03** | 1.81±0.16** |
|  | AA+HS | 1.49±0.07^##^ | 1.13±0.13^##^ | 1.22±0.12^##^ |
| + Snail | Control | 1.22±0.12 | 0.8±0.03 | 1.19±0.0.04 |
|  | AA | 2.48±0.13** | 0.56±0.07** | 1.79±0.12** |
|  | AA+HS | 2.48±0.19** | 0.58±0.02** | 1.86±0.19** |

***P*<0.01 vs. control group, ^##^*P*<0.01 vs. AA alone.
