# Supplementary material for: Hirsutella sinensis Attenuates Aristolochic Acid-Induced Renal Tubular Epithelial-Mesenchymal Transition by Inhibiting TGF-β1 and Snail Expression
Source: PLoS One. 2016 Feb 18;11(2):e0149242. doi: 10.1371/journal.pone.0149242 (PMC4759455; doi:10.1371/journal.pone.0149242)
Supplement: S1 Table — (DOC) [file pone.0149242.s002.doc]

**Table 1 Primer sequences for real time quantitative RT-PCR analysis**

**I**n animal experiment

| **Target** |  | **Primer sequence (5’-3’)** |
| --- | --- | --- |
| **TGF-β1** | **Forward** | **CGGCAGCTGTACATTGACTTT** |
|  | **Reverse** | **TGTGTTGGTTGTAGAGGGCAA** |
| **Snail** | **Forward** | **TTCACATCCGAGTGGGTCTG** |
|  | **Reverse** | **ACCCACACTGGTGAGAAGCC** |
| **α-SMA** | **Forward** | **TCCAGAGCGACATAGCACAG** |
|  | **Reverse** | **CCGAGATCTCACCGACTACC** |
| **Cytokeratin-18** | **Forward** | **GCCAGACCCAGGAATACGA** |
|  | **Reverse** | **CCACTTTGCCATCCACGAC** |
| **GAPDH** | **Forward** | **TGGGTGTGAACCACGAGAA** |
|  | **Reverse** | **GGCATGGACTGTGGTCATGA** |
