# Supplementary material for: Hirsutella sinensis Attenuates Aristolochic Acid-Induced Renal Tubular Epithelial-Mesenchymal Transition by Inhibiting TGF-β1 and Snail Expression
Source: PLoS One. 2016 Feb 18;11(2):e0149242. doi: 10.1371/journal.pone.0149242 (PMC4759455; doi:10.1371/journal.pone.0149242)
Supplement: S2 Table — (DOC) [file pone.0149242.s003.doc]

**Table 2 Primer sequences for real time quantitative RT-PCR analysis**

in cell experiment

| **Target** |  | **Primer sequence (5’-3’)** |
| --- | --- | --- |
| **TGF-β1** | **Forward** | **GGAGCAATGATCTTGATCTTC** |
|  | **Reverse** | **CCTTCCTGGGCATGGAGTCCTG** |
| **Snail** | **Forward** | **ACCCCAATCGGAAGCCTAACT** |
|  | **Reverse** | **GGTCGTAGGGCTGCTGGAA** |
| **α-SMA** | **Forward** | **GGGACGACATGGAAAAGATCTG** |
|  | **Reverse** | **CAGGGTGGGATGCTCTTCAG** |
| **Cytokeratin-18** | **Forward** | **GCTGGAAGATGGCGAGGAC** |
|  | **Reverse** | **TTTATTGGCCTCCTGCTCCC** |
| **β-actin** | **Forward** | **GGAGCAATGATCTTGATCTTC** |
|  | **Reverse** | **CCTTCCTGGGCATGGAGTCCTG** |
