# Supplementary material for: Hirsutella sinensis Attenuates Aristolochic Acid-Induced Renal Tubular Epithelial-Mesenchymal Transition by Inhibiting TGF-β1 and Snail Expression
Source: PLoS One. 2016 Feb 18;11(2):e0149242. doi: 10.1371/journal.pone.0149242 (PMC4759455; doi:10.1371/journal.pone.0149242)
Supplement: S3 Table — (DOC) [file pone.0149242.s004.doc]

Table 3 Primary and secondary antibodies for Western blot assay

| **Primary antibody** | **Secondary antibody** |
| --- | --- |
| **Rabbit anti-TGF-β1 pAb**  **(Santa Cruz)** | **FITC-labeled goat anti-rabbit IgG antibody (Dako)** |
| **Rabbit anti-Snail pAb**  **(Cell Signaling)** | **Ditto** |
| **Rabbit anti-cytokeratin 18 mAb**  **(Cowin Biotech)** | **Ditto** |
| **Mouse anti-α-SMA mAb**  **(Cowin Biotech)** | **FITC-labeled goat anti-mouse IgG antibody (Dako)** |
| **Mouse anti-β-actin mAb**  **(Santa Cruz)** | **Ditto** |
